# Perioperative Chemo-Immunotherapy in Non-Oncogene-Addicted Resectable Non-Small Cell Lung Cancer (NSCLC): Italian Expert Panel Meeting

**DOI:** 10.3390/curroncol32020110

**Published:** 2025-02-14

**Authors:** Filippo de Marinis, Andrea Ardizzoni, Ilaria Attili, Laura Bonanno, Emilio Bria, Diego Luigi Cortinovis, Stefano Margaritora, Francesca Mazzoni, Edoardo Mercadante, Alessandro Morabito, Francesco Petrella, Federico Rea, Rosario Salvi, Piergiorgio Solli, Lorenzo Spaggiari, Luca Voltolini, Cesare Gridelli

**Affiliations:** 1 Division of Thoracic Oncology, European Institute of Oncology, IRCCS, 20141 Milan, Italy; ilaria.attili@ieo.it; 2 Division of Medical Oncology, IRCCS Azienda Ospedaliero-Universitaria di Bologna, 40138 Bologna, Italy; andrea.ardizzoni2@unibo.it; 3 Department of Experimental, Diagnostic and Specialty Medicine (DIMES), University of Bologna, 40126 Bologna, Italy; 4 Department of Surgery, Oncology and Gastroenterology, University of Padova, 35122 Padova, Italy; laura.bonanno@iov.veneto.it; 5 Medical Oncology 2, Veneto Institute of Oncology IOV, IRCCS, 35128 Padova, Italy; 6 Fondazione Policlinico Universitario Agostino Gemelli IRCCS, Università Cattolica del Sacro Cuore, 00136 Rome, Italy; emilio.bria@unicatt.it; 7 Ospedale Isola Tiberina–Gemelli Isola, 00186 Rome, Italy; 8 Medical Oncology, Fondazione IRCCS San Gerardo dei Tintori, 20900 Monza, Italy; diegoluigi.cortinovis@irccs-sangerardo.it; 9 Department of Medicine, Università Milano-Bicocca, 20126 Milano, Italy; 10 Thoracic Surgery, Fondazione Policlinico Universitario Agostino Gemelli IRCCS, 00136 Rome, Italy; stefano.margaritora@policlinicogemelli.it; 11 Medical Oncology Unit, Careggi University Hospital, 50134 Florence, Italy; mazzonifr@aou-careggi.toscana.it; 12 Thoracic Surgery, Istituto Nazionale Tumori “Fondazione G. Pascale”-IRCCS, 80131 Napoli, Italy; edoardo.mercadante@istitutotumori.na.it; 13 Thoracic Medical Oncology, Istituto Nazionale Tumori “Fondazione G. Pascale”-IRCCS, 80131 Napoli, Italy; alessandromorabito1@virgilio.it; 14 Division of Thoracic Surgery, Fondazione IRCCS San Gerardo dei Tintori, 20900 Monza, Italy; francesco.petrella@irccs-sangerardo.it; 15 Thoracic Surgery Unit, Department of Cardiologic, Thoracic and Vascular Sciences, University Hospital, 35128 Padova, Italy; federico.rea@unipd.it; 16 Division of Thoracic Surgery, S.G. Moscati Hospital, 83100 Avellino, Italy; rosario.salvi@aornmoscati.it; 17 Division of Thoracic Surgery, Fondazione IRCCS Istituto Nazionale dei Tumori, 20133 Milan, Italy; piergiorgio.solli@istitutotumori.mi.it; 18 Division of Thoracic Surgery, European Institute of Oncology, IRCCS, 20141 Milan, Italy; lorenzo.spaggiari@ieo.it; 19 Department of Oncology and Hemato-Oncology, University of Milan, 20141 Milan, Italy; 20 Department of Experimental and Clinical Medicine, University of Florence, 50121 Florence, Italy; luca.voltolini@unifi.it; 21 Thoracic Surgery Unit, Careggi University Hospital, 50134 Florence, Italy; 22 Division of Medical Oncology, S.G. Moscati Hospital, 83100 Avellino, Italy; cgridelli@libero.it

**Keywords:** surgery, pembrolizumab, durvalumab, nivolumab, atezolizumab, early stage, NSCLC

## Abstract

Background: Immunotherapy (IO)-based strategies have been demonstrated to significantly prolong survival in the perioperative setting of non-oncogene-addicted non-small cell lung cancer (NSCLC). The adoption of such strategies in clinical practice depends on heterogeneous regulatory approvals and on the agreement between medical oncologists and thoracic surgeons on patients’ selection. Methods: An Expert Panel Meeting of medical oncologists and thoracic surgeons was held virtually by the Italian Association of Thoracic Oncology (AIOT) to discuss results of pivotal clinical trials with perioperative chemo-immunotherapy and reach agreement on open issues for the topic, formulating specific statements based on initially proposed discussion questions. Results: Overall, panelists found agreement on seven statements. With regard to tissue and biomarker analysis, the role of increasing PD-L1 expression in predicting IO efficacy was recognized, whereas ctDNA and pCR were mainly attributed a prognostic role, in the absence of dedicated studies. The panelists acknowledged direct relationship between the benefit of neoadjuvant chemo-immunotherapy approaches and the local burden of disease/mediastinal node involvement, supporting the inclusion of these factors, together with PD-L1, in selecting upfront surgery or induction treatment. The panelists agreed that the current literature data do not answer the issue of assessing the role of the adjuvant phase within a perioperative treatment strategy. Surgical considerations on the role of pneumonectomy and other approaches were also discussed. Conclusions: This experience highlights the importance of a synergistic approach between oncologists and surgeons to leverage the unmet needs in translating results of IO-perioperative clinical trials into clinical practice in patients with resectable NSCLC.

## 1. Introduction

Non-small cell lung cancer (NSCLC) represents the majority of lung cancer cases worldwide and remains a leading cause of cancer-related mortality [1]. Despite the curative intent of surgery in early-stage settings, recurrence rates following surgical resection remain high, particularly in patients with stage II and III disease [2,3,4]. Traditional perioperative strategies, such as neoadjuvant or adjuvant chemotherapy, have provided only modest survival benefits [3,5,6].

The advent of immune checkpoint inhibitors (ICIs), targeting programmed death-1 (PD-1) and programmed death-ligand 1 (PD-L1), has revolutionized cancer therapy, demonstrating durable responses in advanced-stage NSCLC [7,8]. Building on this success, immunotherapy (IO) is now being integrated into the perioperative setting, including neoadjuvant and adjuvant immunotherapy, to enhance outcomes in resectable NSCLC by leveraging the host immune system to eradicate micrometastatic disease, induce immunologic memory, and reduce recurrence risk [9,10,11].

Pivotal phase III studies evaluating the role of immune checkpoint inhibitors in the perioperative setting of resectable NSCLC include CheckMate 816 [12,13] (neoadjuvant nivolumab plus chemotherapy versus chemotherapy alone), Keynote-671 [14,15], AEGEAN [16], CheckMate 77T [17] (respectively, pembrolizumab, durvalumab, and nivolumab, combined with neoadjuvant chemotherapy and continued as adjuvant monotherapy), IMpower010 [18,19] (atezolizumab following adjuvant chemotherapy in resected stage II-IIIA NSCLC), and Keynote-091 [20] (pembrolizumab versus placebo after resection and optional chemotherapy in NSCLC). Other similar studies were conducted in the Asian population [21,22,23]. All of these strategies demonstrated significantly prolonged event-free survival (EFS) or disease-free survival (DFS), compared to the previous standard approach of chemotherapy alone, and are at different steps of their international regulatory approvals by the Food and Drug Administration (FDA), the European Medicines Agency (EMA), and the Agenzia Italiana del Farmaco (AIFA) (Table 1), depicting heterogeneous possible future scenarios of applicability [11].

This paper aims to integrate the perspectives of medical oncologists and thoracic surgeons on the use of novel perioperative IO-based treatments in NSCLC, emphasizing recent clinical evidence, the integration of biomarkers, and the potential for improving long-term outcomes in surgically resectable disease in clinical practice.

## 2. Materials and Methods

An Expert Panel Meeting was held virtually by the Italian Association of Thoracic Oncology (AIOT) on 8 October 2024. Eight thoracic surgeons and nine medical oncologists from different Italian regions composed the panel.

During the first part of the meeting, the results of pivotal clinical trials with perioperative chemo-immunotherapy were reviewed to point out analogies and differences in methodology, results, and subgroups.

The second part of the meeting was used to reach common statements on open issues for the topic, with three subject areas identified: (1) tissue and biomarker analysis; (2) chemo-immunotherapy approaches to treat resectable NSCLC; and (3) surgical considerations.

Key questions on each subject area, previously agreed upon by 3 panelists (FdM, IA, and CG), were proposed with a live electronic voting system used to stimulate discussion (Supplementary Figure S1).

Live minutes were used to collect the panelists’ shared statements, which served as the basis to build up the current manuscript.

## 3. Perioperative Options in Non-Oncogene-Addicted Resectable NSCLC

The CheckMate 816 trial established the benefit of neoadjuvant nivolumab plus chemotherapy, showing improved pathologic complete response (pCR) rates (24% vs. 2.2%) and event-free survival (EFS) compared to chemotherapy alone [12] (Table 2 and Table 3). Keynote-671 evaluated pembrolizumab in neoadjuvant and then adjuvant settings, demonstrating significant improvement in EFS and overall survival (OS) [14,15] (Supplementary Table S1). The AEGEAN trial confirmed the efficacy of perioperative durvalumab with chemotherapy, achieving enhanced EFS compared to chemotherapy alone [16].

The CheckMate 77T study also assessed nivolumab in a perioperative approach, with EFS HR 0.58 (97% CI 0.42–0.81) [17] (Table 2 and Table 3).

In the adjuvant setting, the IMpower010 trial showed that atezolizumab after adjuvant chemotherapy prolonged DFS and OS in patients with PD-L1-positive stage II-IIIA NSCLC (DFS HR 0.70, 95% CI 0.55–0.91 in stage II-IIIA PD-L1 ≥ 1%; DFS HR 0.48, 95% CI 0.32–0.72 in stage II-IIIA PD-L1 ≥ 50%; OS HR 0.71 in PD-L1 ≥ 1%; HR 0.43 in PD-L1 ≥ 50%) [18,19]. Conversely, Keynote-091 revealed pembrolizumab’s DFS benefit compared to placebo in a population with resected NSCLC, regardless of PD-L1 expression [20].

Several trials focusing on ICIs developed in China are further expanding the evidence base. RATIONALE 315 investigated neoadjuvant tislelizumab plus chemotherapy in resectable NSCLC, demonstrating early safety and efficacy signals [22]. ORIENT-31 and related studies are evaluating sintilimab, a PD-1 inhibitor, in perioperative combinations, aiming to confirm its utility in improving DFS and OS [23]. Neotorch explored neoadjuvant toripalimab with chemotherapy, showing favorable pCR rates and safety, with ongoing trials aiming to validate long-term outcomes [21].

## 4. Open Issues on Perioperative Treatments in NSCLC

Based on the overall results, open issues were identified and categorized in three subject areas. The first issue was related to tissue and biomarker analysis, and included discussion of PD-L1, circulating tumor DNA (ctDNA), pathologic complete response (pCR), and molecular testing. The second big topic aimed at discussing the choice among the available immunotherapy-based approaches to treat resectable NSCLC. Finally, the third issue involved surgical considerations for non-oncogene-addicted resectable NSCLC.

### 4.1. Tissue and Biomarker Analysis

#### 4.1.1. PD-L1

The use of PD-L1 as a predictive biomarker of response to ICIs in non-oncogene-addicted advanced NSCLC [27] led to exploring its role in the early-stage NSCLC setting as well. Indeed, PD-L1 was used as a stratification factor, although with different cut-offs, in all pivotal trials with perioperative immunotherapy-based strategies (Table 2). With the exception of the IMpower 010 study of adjuvant atezolizumab with a DFS hierarchical testing design according to PD-L1 and stage [18], the study survival endpoints were positive in the overall populations in all of the considered trials. However, subgroup analyses show differential magnitudes of benefits according to PD-L1 values (Table 3).

Hence, two discussion questions (Q1–Q2) were proposed to the panelists to initiate discussion on the role of PD-L1 in clinical practice in the early-stage NSCLC setting:

Q1. ‘Looking at the next regulatory reimbursements by AIFA in Italy, and considering the results of the subgroup analysis in the CheckMate 816 trial [12], with differential approval by FDA and EMA, do you agree to exclude PD-L1 negative patients from receiving Checkmate 816 regimen?’ (Yes: 29%; No: 79%; voting: 17/17).
Q2. ‘Looking at the next regulatory reimbursements by AIFA in Italy for the Keynote-671 regimen, and considering the OS (study coprimary endpoint) subgroup analysis [15], do you agree on the FDA and EMA approvals regardless of PD-L1?’ (Yes: 81%; No: 19%; voting: 17/17).

The following statement (Statement 1) was then formulated by the panelists on this issue:

**Which is the role of PD-L1 in resected NSCLC (*EGFR* and *ALK* wt)?**

**STATEMENT 1:**

PD-L1 has a predictive role for ICI efficacy, in terms of benefit that is proportional to increasing PD-L1 values, consistent across endpoints and across studies. However, negative PD-L1 value is not considered an excluding factor for immunotherapy-based perioperative treatments.

#### 4.1.2. Pathologic Complete Response (pCR) and Circulating Tumor DNA (ctDNA)

In the context of resected NSCLC, pCR refers to the absence of residual viable cancer cells in the primary tumor and sampled lymph nodes following neoadjuvant treatments, as determined by histopathological examination after surgical resection [28].

ctDNA refers to fragments of DNA that are shed into the bloodstream by tumor cells. It can be detected and analyzed through blood-based liquid biopsies in patients with NSCLC, and is used to track tumor dynamics, treatment response, and potential recurrence or progression [29,30]. In resected NSCLC settings, the detection of ctDNA after surgery—Minimal Residual Disease (MRD)—may indicate residual cancer and risk of relapse [31,32].

Both pCR (coprimary endpoint in CheckMate 816 and AEGEAN trials) and ctDNA have been investigated as pivotal biomarkers in IO-perioperative trials: achieving pCR, as well as the absence of post-surgical ctDNA, are associated with improved long-term outcomes [13,24,25,26] (Table 3).

The following two questions (Q3–Q4) were used to begin the discussion:

Q3. ‘Pathologic response (pCR or not pCR) to chemo-immunotherapy has a role in the choice to adopt or not subsequent adjuvant immunotherapy?’ In this case, three options were proposed: I would not administer adjuvant immunotherapy after pCR but I would do in the absence of pCR (31%); I would administer adjuvant immunotherapy after pCR but I would not do in the absence of pCR (13%); No (56%) (voting: 16/17; abstained: 1/17).
Q4. ‘Based on results obtained in clinical trials, should ctDNA evaluation, if reimbursed, be used to guide the choice to adopt or not subsequent adjuvant immunotherapy (regardless of previous neoadjuvant treatment)?’ (Yes, I would administer adjuvant immunotherapy in ctDNA-positive and not in ctDNA-negative situations: 56%; No: 44%; voting: 16/17; abstained: 1/17).

Panelists expressed their agreement in the following, Statements 2 and 3:

**Which is the role of pCR in resected NSCLC (*EGFR* and *ALK* wt)?**

**STATEMENT 2:**

Clinical trials have not been designed to evaluate the differential impact of administering or not adjuvant immunotherapy based on pCR confirmation. Hence, only a prognostic role of pCR can be considered, to date. This information should be considered, together with ctDNA, in dedicated studies.

**Which is the role of ctDNA in resected NSCLC (*EGFR* and *ALK* wt)?**

**STATEMENT 3:**

Clinical trials have not been designed to evaluate the differential impact of administering or not adjuvant immunotherapy based on the presence/absence of post-surgical ctDNA. Hence, only a prognostic role of ctDNA can be considered, to date. This information should be considered, together with pCR, in dedicated studies.

#### 4.1.3. Molecular Testing

*EGFR*- and *ALK*-positive patients were excluded in the pivotal trials with perioperative chemo-immunotherapy, either as an exclusion criteria for study entry or as excluded from the primary analysis results [2]. Hence, IO-based treatments are not indicated in this setting. Furthermore, targeted options are available with tyrosine kinase inhibitors in the adjuvant setting for *EGFR*-mutant and ALK-rearranged disease, so the pre-surgical molecular status of these two genes is crucially needed [2,33,34,35].

Two initial questions (Q5–Q6) were proposed to the panelists on the role of molecular testing in the early-stage setting.

Q5. ‘Based on results obtained with IO-based treatments in the advanced stage in patients with NSCLC harboring driver oncogene alterations, should pre-operative molecular testing be limited to *EGFR* and *ALK* evaluation, or is it necessary to adopt a wider molecular panel testing?’ (only *EGFR* and *ALK*: 35%; wider molecular panel: 65%; voting 17/17).
Q6. ‘Based on results obtained with IO-based treatments in the advanced stage in patients with NSCLC harboring driver oncogene alterations [36,37,38], do you believe that being aware of the presence of such alterations (besides *EGFR* and *ALK*) before surgery could impact the choice of IO-based neoadjuvant/adjuvant/perioperative treatments?’ (Yes: 100%; No: 0%; voting: 17/17).

Agreement was reached in the following, Statement 4:

**Which is the role of molecular testing in resectable NSCLC?**

**STATEMENT 4:**

Based on results obtained in pivotal trials, pre-surgical molecular testing should include at least *EGFR* and *ALK* status evaluation, however extended next generation sequencing (NGS) panel would be preferred. Indeed, although this was not addressed in clinical trials, the information would be helpful to evaluate excluding from IO-based strategies patients with other driver oncogene alterations which are known to be poor responder to immunotherapy treatments.

### 4.2. Chemo-Immunotherapy Approaches to Treat Resectable NSCLC

#### 4.2.1. Choosing Between Upfront Surgery or Induction Treatments

In the presence of equally available regimens for resectable NSCLC, one of the most important issues in clinical practice is the choice between upfront surgery or induction treatments, to be agreed between clinicians and surgeons in a common view [10,39,40].

Two discussion questions (Q7–Q8) were proposed to initiate discussion on this topic.

Q7. ‘In your future clinical practice, considering current and future regulatory approvals, results of clinical trials in the perioperative setting should be interpreted only for global results or also taking into account subgroup analysis?’ (only global results: 25%; also subgroups (stage, nodal status, PD-L1): 75%; voting: 17/17).
Q8. ‘Based on subgroup analyses results by stage and N, and the nearly 20% of patients not undergoing surgery after induction in clinical trials [12,15,16,17], do you believe there is any preferred indication for neoadjuvant chemo-immunotherapy versus upfront surgery followed by adjuvant treatment?’ (Yes: 69%; No: 31%; voting 17/17).

The panelists then found agreement in the following, Statement 5:

**Which is the role of clinical stage, nodal status, PD-L1, in the choice between upfront surgery (eventually followed by adjuvant immunotherapy) and perioperative treatment?**

**STATEMENT 5:**

Although based on subgroup analyses, the benefit from immunotherapy appears greater in stage III compared to stage II, in N2 involvement compared to N0-N1, in PD-L1 positive compared to negative patients. These factors can be considered, in clinical practice, for preferring patients candidate for perioperative chemo-immunotherapy.

A role for adjuvant chemotherapy followed by immunotherapy has been also demonstrated in resected NSCLC with PD-L1 ≥ 50% [19], so that upfront surgery option can be considered in such cases.

#### 4.2.2. Choosing Between Neoadjuvant Strategy Alone Versus Neoadjuvant Followed by Adjuvant Strategy

Four questions (Q9–Q12) were proposed for the specific issue of choosing between exclusive neoadjuvant or perioperative (neoadjuvant followed by adjuvant) strategies, based on key results available from clinical trials (Table 2 and Table 3).

Q9. ‘Based on the differences observed within stage and N subpopulations in each clinical trials [12,15,16,17], do you think this can influence the choice of the specific PD-1/PD-L1 inhibitor in stage II?’ (Yes: 47%; No: 53%; voting: 15/17; abstained: 2/17).
Q10. ‘How to choose upfront between neoadjuvant chemo-immunotherapy (CheckMate 816 regimen) and perioperative chemo-immunotherapy (Keynote-671, CheckMate 77T, Aegean regimens) [12,15,16,17]?’ For this question, three possible factors were considered, and panelists voted according to priority (possibility to adopt following adjuvant immunotherapy: 93%; patients’ clinical factors: 7%; planned surgery type: 0%; voting 15/17; abstained: 2/17).
Q11. Considering results obtained with different platinum salt used [12,15,16,17], do you think cisplatin should be preferred, in the absence of clinical contraindications? (Yes: 14%; No: 86%; voting: 14/17; abstained: 3/17).
Q12. Considering results presented on patients who received or not adjuvant phase in the AEGEAN trial [25] and the post-surgery landmark results of CheckMate 77T vs. CheckMate 816 (DFS HR 0.61, 95% CI 0.39–0.97) [41], do you believe there is a rationale not to administer adjuvant phase after neoadjuvant chemo-immunotherapy, to date? (Yes: 20%; No: 80%; voting: 15/17; abstained: 2/17).

Statement 6 was then agreed, as follows:

**Which factors to be considered in the choice between exclusive neoadjuvant vs. perioperative chemo-immunotherapy?**

**STATEMENT 6:**

To date, prospective studies designed to compare neoadjuvant alone vs. perioperative regimens are not available. In the absence of such studies, there are no elements to prefer in advance the one or the other approach.

### 4.3. Surgical Considerations

Three practical questions were proposed for the third issue, on surgical considerations in the perioperative setting.

The first question (Q13) was ‘Planned surgery–pneumonectomy vs. non-pneumonectomy–has a role in the choice whether to candidate or not a patient to neoadjuvant chemo-immunotherapy?’ (Yes: 71%; No: 29%; voting: 17/17).

The second question (Q14) was ‘Planned surgery has a role in the choice between 3 or 4 cycles of neoadjuvant chemo-immunotherapy?’ (Yes: 18%; No: 82%; voting: 17/17).

The third question (Q15) was ‘Taking into account the available options in the neo- and adjuvant setting, is there still a role for anatomic sub-lobar resections?’ (Yes: 65%; No: 35%; voting: 17/17).

Hence, the final statement (Statement 7) included shared considerations for the role of surgical strategies to be integrated with systemic treatments in clinical practice, as follows:

**Which is the role of planned surgery in the choice among available IO peri-operative options?**

**STATEMENT 7:**

In clinical practice, risk of pneumonectomy should be carefully assessed preoperatively, and it is a relevant element in preferring upfront surgery rather than a neoadjuvant strategy, with a tendency favoring the latter although planned pneumonectomy was excluded in some neoadjuvant clinical trials

Broncho-vascular lung-sparing procedures should be preferred and pursued in any circumstances in order to prevent the option of pneumonectomy [42,43].

The number of neoadjuvant treatment cycles is not dependent on the planned surgery type.

Anatomical segmentectomies could be also considered after chemo-immunotherapy, only following a careful selection of cases and providing that all the oncological principles of an R0 resection are matched [44].

## 5. Conclusions

**Figure 1:**
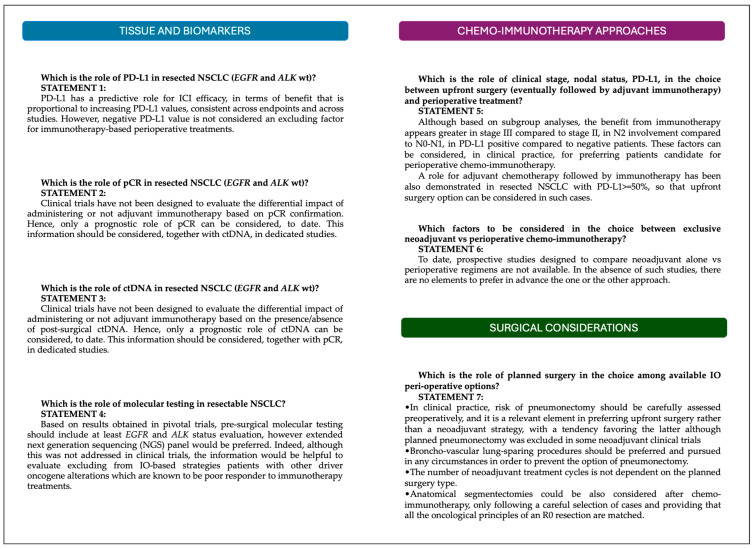
Summary of the consensus statements.

The integration of immune checkpoint inhibitors (ICIs) into perioperative management represents a significant advance in the treatment of resectable NSCLC, offering the potential to improve long-term survival outcomes and reduce recurrence rates. During the Italian Expert Panel Meeting, medical oncologists and thoracic surgeons found agreement on seven statements aiming to leverage the unmet needs in translating clinical trials results into clinical practice (Figure 1).

Notably, the collection of the responses from the voting system was anonymous with the aim of allowing for spontaneous replies unaffected by roles and of stimulating discussion. As such, it was not possible to distinguish between oncologists’ and surgeons’ answers. However, the lack of initial consensus on many of the questions greatly reflects the imperfect and incomplete level 1 evidence that is available on the topic.

By fostering a synergistic approach between oncologists and surgeons, this experience highlights the importance of multidisciplinary collaboration to optimize the use of these novel therapies with the goal of establishing evidence-based, patient-centered pathways that improve survival while maintaining quality of life in patients with resectable NSCLC. This collaboration will be essential in translating the promising results of clinical trials into real-world practice.

## Figures and Tables

**Table 1:** Current regulatory approval status of immunotherapy-based regimens in the perioperative setting.

	Checkmate 816 [12]	Keynote-671 [15]	Aegean [16]	Checkmate 77T [17]	Impower-010 [19]	Keynote-091 [20]
FDA	PD-L1 all-comers (2022)	PD-L1 all-comers (2023)	PD-L1 all-comers ^§^ (2024)	PD-L1 all-comers ^§^ (2024)	PD-L1 ≥ 1% (2021)	PD-L1 all-comers (2023)
EMA	PD-L1 ≥ 1% (2023)	PD-L1 all-comers (2024)	-	-	PD-L1 ≥ 50% ^§^ (2022)	PD-L1 all-comers (2023)
AIFA	-	-	-	-	PD-L1 ≥ 50% ^§^ (2023)	-

^§^ *EGFR* and *ALK* excluded.

**Table 2:** Main study design characteristics and study populations across perioperative chemo-immunotherapy trials.

Trial	Checkmate 816 [12]	Keynote-671 [14]	Aegean [16]	Checkmate 77T [17]
Timing	Neo	Neo → Adj	Neo → Adj	Neo → Adj
Design	Open label	Double blind	Double blind	Double blind
ICI agent	Nivolumab	Pembrolizumab	Durvalumab	Nivolumab
Chemo agent	Platinum-doublet	Cisplatin doublet	Platinum-doublet	Platinum-doublet
% use of cisplatin *	69%	100%	27%	24%
cycles of ICI	3 (neo)	4 (neo) + 13 (adj)	4 (neo) + 12 (adj)	4 (neo) + 12 (adj)
Inclusion	Resectable IB (≥4 cm)-IIIA (7th)	Resectable II-IIIB (8th)	Resectable II-IIIB (8th)–excl. pneumonectomy	Resectable II-IIIB (8th)
Distribution by stage (clinical) *	IB-II: 36% III: 63%	II: 30% IIIA: 55% IIIB: 15%	II: 28% IIIA: 47% IIIB: 24%	II: 35% III: 65%
Distribution by N *	pN−: 47% pN+: 53%	cN0: 37% cN1: 20% cN2: 42%	cN0: 30% cN1: 21% cN2: 49%	cN0: 35% cN1: 25% cN2: 40%
Primary endpoint	EFS; pCR	EFS; OS	EFS; pCR	EFS
Stratification factors	Stage: IB-II vs. III PD-L1: <1%, ≥1% Sex	Stage: II vs. III PD-L1: <50%, ≥50%	Stage: II vs. III PD-L1: <1%, ≥1%	Stage: II vs. III PD-L1: <1%, ≥1%, ne Histo: Sq vs. non-sq

* Data refer to experimental arm. Data are presented with rounding. Abbreviations: adj: adjuvant; cN: clinical nodal status; EFS: event-free survival; ICI: immune checkpoint inhibitor; neo: neoadjuvant; pCR: pathologic complete response; pN: pathologic nodal status; OS: overall survival.

**Table 3:** Main EFS results across perioperative chemo-immunotherapy trials.

Trial	Checkmate 816 [12,13]	Keynote-671 [14,15,24]	Aegean [16,25]	Checkmate 77T [17,26]
Median follow up (months) ^a^	57	36.6	25.9	33.3
EFS months, HR (95% CI)	43.8 vs. 18.4 HR 0.66 (0.49–0.90)	47.2 vs. 18.3 HR 0.59 (0.48–0.72)	NR vs. 30 HR 0.69 (95% CI 0.55–0.88)	NR vs. 18.4 HR 0.58 (97% CI 0.42–0.81)
EFS rate	24 m: 63.8% vs. 45.3% 48 m: 49% vs. 38%	24 m: 62.4% vs. 40.6% 48 m: 48.4% vs. 26.2%	24 m: 63.3% vs. 52.4% 36 m: 60.1% vs. 47.9%	18 m: 70.2% vs. 50%
EFS by PD-L1 HR (95% CI)	<1%: 0.85 (0.54–1.32) ≥1%: 0.41 (0.24–0.70) ≥50%: 0.24 (0.10–0.61)	<1%: 0.75 (0.56–1.01) 1–49%: 0.52 (0.36–0.73) ≥50%: 0.48 (0.33–0.71)	<1%: 0.76 (0.49–1.17) 1–49%: 0.70 (0.46–1.05) ≥50%: 0.60 (0.35–1.01)	<1%: 0.73 (0.47–1.15) ≥1%: 0.52 (0.35–0.78) ≥50%: 0.26 (0.12–0.55)
EFS by Stage HR (95% CI)	IB-II: 0.87 (0.48–1.56) III: 0.54 (0.37–0.80)	II: 0.59 (0.40–0.88) IIIA: 0.57 (0.44–0.74) IIIB: 0.57 (0.36–0.93)	II: 0.76 (0.43–1.34) IIIA: 0.57 (0.39–0.83) IIIB: 0.83 (0.52–1.32)	II: 0.81 (0.46–1.43) III: 0.51 (0.36–0.72)
EFS by N HR (95% CI)	pN−: 0.74 (0.39–1.41) pN+: 0.69 (0.42–1.13)	N0: 0.58 (0.41–0.81) N1: 0.56 (0.35–0.91) N2: 0.63 (0.48–0.82)	N2 single: 0.61 (0.39–0.94) N2 multi: 0.69 (0.33–1.38)	N0: 0.80 (0.48–1.32) N1: 0.58 (0.29–1.16) N2: 0.46 (0.30–0.70)
EFS by Chemo HR (95% CI)	CDDP: 0.71 (0.49–1.03) CBDCA: 0.31 (0.14–0.67)	-	CDDP: 0.58 (0.35–0.93) CBDCA: 0.75 (0.57–0.97)	CDDP: 0.61 (0.35–1.08) CBDCA: 0.53 (0.37–0.75)
pCR	24% vs. 2.2%	18.1% vs. 4%	17.2% vs. 4.3%	25.3% vs. 4.7%
EFS by pCR HR (95% CI)	pCR: NC no pCR: 0.89 (0.64–1.22)	pCR: 0.33 (0.09–1.22) no pCR: 0.69 (0.55–0.85)	pCR: 0.31 (0.07–1.51) no pCR: 0.82 (0.58–1.15)	pCR: 0.32 (0.08–1.35) no pCR: 0.73 (0.49–1.09)

^a^. Longest follow up presented. Not all results reported referred to the latest follow up. Abbreviations: CBDCA: carboplatin; CDDP: cisplatin; EFS: event-free survival; HR: hazard ratio; ICI: immune checkpoint inhibitor; N: nodal status; NR: not reached; pCR: pathologic complete response; pN: pathologic nodal status.

## Data Availability

The original contributions presented in this study are included in the article/Supplementary Material. Further inquiries can be directed to the corresponding author(s).

## Supplementary Materials

The following supporting information can be downloaded at https://www.mdpi.com/article/10.3390/curroncol32020110/s1, Figure S1: Discussion questions for the panelists; Table S1: Main secondary and exploratory results across perioperative chemo-immunotherapy trials.
