# Supplementary material for: Perioperative Chemo-Immunotherapy in Non-Oncogene-Addicted Resectable Non-Small Cell Lung Cancer (NSCLC): Italian Expert Panel Meeting
Source: Curr Oncol. 2025 Feb 14;32(2):110. doi: 10.3390/curroncol32020110 (PMC11854886; doi:10.3390/curroncol32020110)
Supplement: Supplementary file 1 [file curroncol-32-00110-s001.zip › curroncol-3463129-supplementary.pdf]

Supplementary Table S1. Main secondary and exploratory results across perioperative chemo-immunotherapy trials

Trial	Checkmate 816 [12,13]	Keynote-671 [14,15,21]	Aegean [16,22]	Checkmate 77T [17,23]
OS	HR 0.71 (0.47-1.07)<br>4-y 71% vs 58%	HR 0.72 (0.56-0.93)<br>36 m: 71% vs 64%	HR 0.89 (0.70-1.14)	-
OS by PD-L1	<1%: 0.81 (0.48-1.36)<br>≥1%: 0.37 (0.20-0.71)	<1%: 0.91 (0.63-1.32)<br>1-49%: 0.69 (0.44-1.07)<br>≥50%: 0.55 (0.33-0.92)	-	-
ctDNA clearance	Prognostic (yes vs no)	-	-Higher rate with durva<br>-correlates with pCR	-higher ctDNA clearance with nivo<br>-higher ctDNA recurrence with PBO
Definitive surgery*	83.2%	79.4%	77.6%	77.7%
Adjuvant phase	11.9% adj chemo	73.2%	66% (completed 68%)	62% (completed 37.1%)

*Similar rates with induction chemotherapy alone

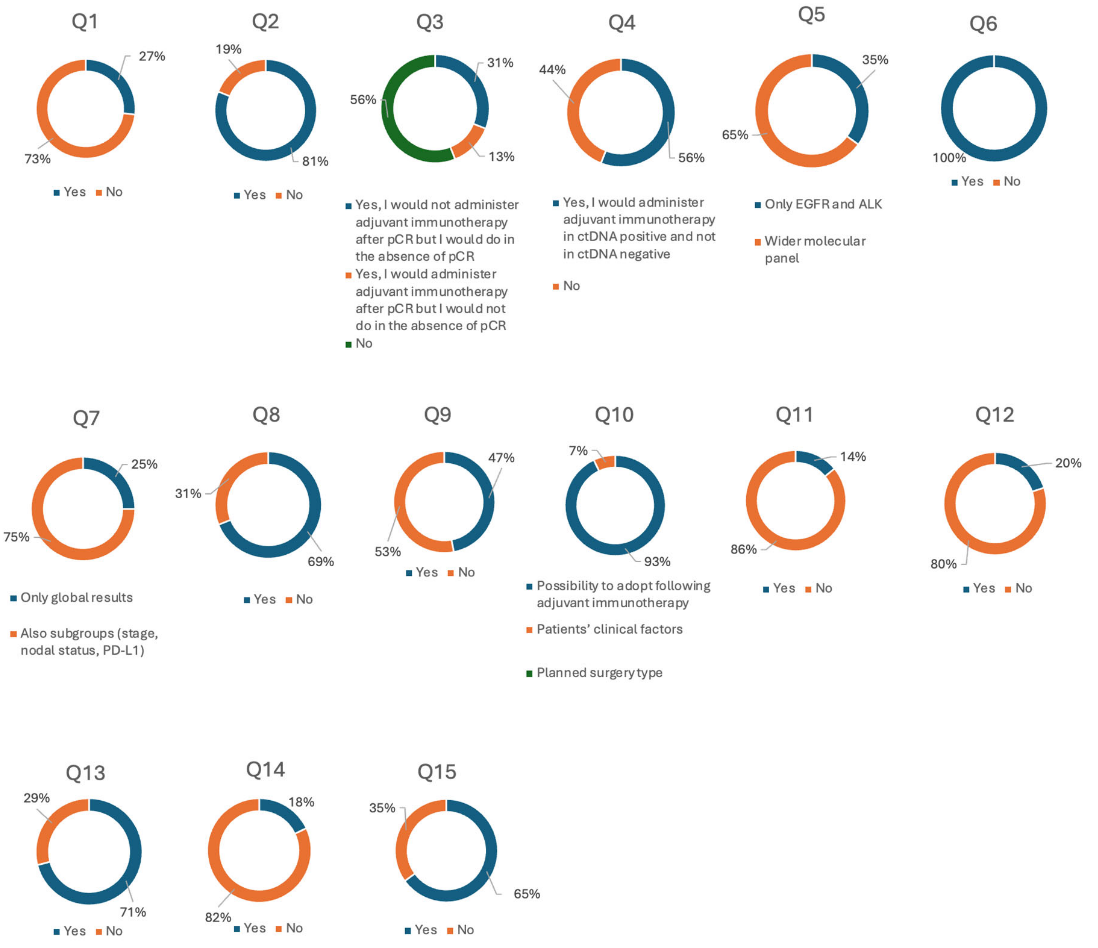

Supplementary Figure S1.
Results of shooting questions' voting:
Q1. Looking at the next regulatory reimbursements by AIFA in Italy, and considering the results of the subgroup analysis in the CheckMate 816 trial, with differential approval by FDA and EMA, do you agree to exclude PD-L1 negative patients from receiving Checkmate 816 regimen?
Q2. Looking at the next regulatory reimbursements by AIFA in Italy for the Keynote-671 regimen, and considering the OS (study coprimary endpoint) subgroup analysis, do you agree on the FDA and EMA approvals regardless of PD-L1?
Q3. Pathologic response (pCR or not pCR) to chemo-immunotherapy has a role in the choice to adopt or not subsequent adjuvant immunotherapy?
Q4. Based on results obtained in clinical trials, should ctDNA evaluation, if reimbursed, be useful to guide the choice to adopt or not subsequent adjuvant immunotherapy (regardless of previous neoadjuvant treatment)?
Q5. Based on results obtained with IO-based treatments in the advanced stage in patients with NSCLC harboring driver oncogene alterations, should pre-operative molecular testing be limited to *EGFR* and *ALK* evaluation, or is it necessary to adopt a wider molecular panel testing?

Q6. Based on results obtained with IO-based treatments in the advanced stage in patients with NSCLC harboring driver oncogene alterations, do you believe that being aware of the presence of such alterations (besides *EGFR* and *ALK*) before surgery could impact the choice of IO-based neoadjuvant/adjuvant/perioperative treatments?
Q7. In your future clinical practice, considering current and future regulatory approvals, results of clinical trials in the perioperative setting should be interpreted only for global results or also taking into account subgroup analysis?
Q8. Based on subgroup analyses results by stage and N, and the nearly 20% of patients not undergoing surgery after induction in clinical trials, do you believe there is any preferred indication for neoadjuvant chemo-immunotherapy versus upfront surgery followed by adjuvant treatment?
Q9. Based on the differences observed within stage and N subpopulations in each clinical trials, do you think this can influence the choice of the specific PD-1/PD-L1 inhibitor in stage II?
Q10. How to choose upfront between neoadjuvant chemo-immunotherapy (CheckMate 816 regimen) and perioperative chemo-immunotherapy (Keynote-671, CheckMate 77T, Aegean regimens)?
Q11. Considering results obtained with different platinum salt used, do you think cisplatin should be preferred, in the absence of clinical contraindications?
Q12. Considering results presented on patients who received or not adjuvant phase in the AEGEAN trial and the post-surgery landmark results of CheckMate 77T vs CheckMate 816 (DFS HR 0.61, 95% CI 0.39-0.97), do you believe there is a rationale not to administer adjuvant phase after neoadjuvant chemo-immunotherapy, to date?
Q13. Planned surgery – pneumonectomy vs non-pneumonectomy – has a role in the choice whether to candidate or not a patient to neoadjuvant chemo-immunotherapy?'
Q14. Planned surgery has a role in the choice between 3 or 4 cycles of neoadjuvant chemo-immunotherapy?
Q15. Taking into account the available options in the neo- and adjuvant setting, is still there a role for sub-lobar resections?
